# Self-efficacy and health-related quality of life: a cross-sectional study of primary care patients with multi-morbidity

**DOI:** 10.1186/s12955-019-1103-3

**Published:** 2019-02-14

**Authors:** Michele Peters, Caroline M. Potter, Laura Kelly, Ray Fitzpatrick

**Affiliations:** Health Services Research Unit, Nuffield Department of Population Health, University of Oxford, Old Road Campus, Oxford, OX3 7LF UK

**Keywords:** Self-efficacy, Quality of life, Multi-morbidity, Primary care

## Abstract

**Background:**

Multi-morbidity in chronic long-term conditions is a major concern for health services. Self-management in concert with clinical care forms part of the effective management of multi-morbidity. Self-efficacy is a mechanism through which self-management can be achieved. Quality of life is adversely impacted by multi-morbidity but could be improved by effective self-management. This study examines the relationship between self-efficacy and quality of life in primary care patients with multi-morbidity.

**Methods:**

A cross-sectional survey was conducted with primary care patients in England. Potential participants were mailed a questionnaire containing quality of life measures (the EQ-5D-5L and the Long-Term Conditions Questionnaire (LTCQ)), the Disease Burden Impact Scale (DBIS) and the Self-efficacy for Managing Chronic Disease Scale. Descriptive statistics, analysis of variance and linear regression analyses were conducted to examine the relationship between quality of life (dependent variable), self-efficacy, and demographic and disease-related variables.

**Results:**

The 848 participants living with multi-morbidity reported a mean of 6.46 (SD 3.49) chronic long-term conditions, with the mean number of physical conditions 5.99 (SD 3.34) and mental health conditions 0.47 (SD 0.66). The mean scores were 15.45 (SD 12.00) for disease burden, 0.69 (SD 0.28) for the EQ-5D-5L, 65.44 (SD 23.66) for the EQ-VAS, and 69.31 (SD 21.77) for the LTCQ. The mean self-efficacy score was 6.69 (SD 2.53). The regression models were all significant at *p* < 0.001 (adjusted R^2^ > 0.70). Significant factors in all models were self-efficacy, disease burden and being permanently sick or disabled. Other factors varied between models, with the most notable being the presence of a mental health condition in the LTCQ model.

**Conclusions:**

Multi-morbid primary care patients with lower self-efficacy and higher disease burden have lower quality of life. Awareness of self-efficacy levels among patients with multi-morbidity may help health professionals identify patients who are in need of enhanced self-management support. Providing self-management support for chronic disease has been hailed as a hallmark of good care. Higher self-efficacy may lead to enhanced quality of life in multi-morbidity.

**Electronic supplementary material:**

The online version of this article (10.1186/s12955-019-1103-3) contains supplementary material, which is available to authorized users.

## Background

Multi-morbidity is a major concern for health services, health research and health policy [1–3]. Strategies and guidelines to manage multi-morbidity are set out by researchers, health policy and governmental bodies internationally [2–4]. Defined as two or more chronic long-term chronic conditions [5], multi-morbidity has been described as the most common chronic condition experienced by adults [6]. Higher use of health services and polypharmacy are more common in people with multi-morbidity than those without [1, 7], making its management complex [8]. Furthermore, multi-morbidity adversely affects patient outcomes such as quality of life and disease burden [7, 9–12]; and impacts on carers, health services and the economy [3].

Due to its increasing prevalence, taking account of multi-morbidity is essential in the design of health services [13] Difficulties with the management of multi-morbidity arise as guidelines and health and care services are often targeted at or organised around single conditions [14, 15]. However, health systems, and professionals working within these, are expected to provide care that is patient-centred and continuous [8], and to support patients to actively self-manage their chronic conditions [4, 15]. Self-management support is highly important in the management of chronic conditions and multi-morbidity [14–16], and health professionals consider it a key vehicle for managing multi-morbidity and reducing service use [14]. Self-management is based on the central premise that individuals need to self-care in a range of health care practices on a day to day basis between medical appointments [17, 18].

Self-efficacy, first defined by Bandura in 1977 [19], refers to the confidence a person has about their capacity to undertake behaviour(s) that may lead to desired outcomes. Self-efficacy is a mechanism through which effective self-management can be achieved [17]. Measuring self-efficacy is a standardised and convenient approach to assess patients’ self-management potential and has been recommended as a component of chronic care management [20]. Marks et al. [21] hypothesised that higher self-efficacy is associated with better outcomes, and that better outcomes reduce health services burden. A meta-analysis concluded that self-management support for a range of single conditions was associated with small but significant improvements in health outcomes, but only a minority of interventions reported reductions in the use of health services [22]. Multi-morbid patients usually find self-management harder [14, 23], for example, because treatments prescribed by different health care providers can lead to conflicts in care across conditions [14]. Self-efficacy can be improved through self-management support, and improvements of the chronic disease outcomes are related to improvements in self-efficacy [24]. Furthermore, it has been shown that higher self-efficacy leads to reduced health care utilization [25].

The assessment of quality of life and self-efficacy have both been identified as part of the core outcome set for multi-morbidity [26]. This paper examines the relationship between self-efficacy and quality of life in primary care patients with multi-morbidity.

## Methods

A cross-sectional postal survey was conducted in primary care in three diverse regions in England (Oxfordshire, North West Coast, Yorkshire & Humber). The main aim of the study was to validate a new measure for long-term conditions (these findings are published elsewhere [27]). The study was reviewed by England’s National Research Ethics Service Committee East Midlands – Derby (reference 15/EM/0414) and approvals were granted by the Health Research Authority of England’s National Health Service (NHS), and local health care organisations linked to participant recruitment sites.

### Recruitment

Potential participants were invited through 15 primary care practices, with the target population being adults (i.e. 18 years of age or above) who had received a diagnosis at least 12 months ago of one of 11 specified chronic conditions: cancer within the last 5 years, chronic back pain, chronic obstructive pulmonary disease (COPD), diabetes, depression, irritable bowel syndrome (IBS), ischaemic heart disease (IHD), multiple sclerosis (MS), osteoarthritis (OA), severe mental health (including psychoses, bipolar disorder and schizophrenia which are the severe mental conditions included in the UK Quality and Outcomes Framework [28]), and stroke. The conditions were selected in an earlier phase of the work [29] to cover a broad range of conditions in terms of their onset, disease burden, trajectory, physiology etc. For conditions with lifelong implications (i.e. COPD, diabetes, IBS, IHD, MS, OA, stroke), participant eligibility was defined as the presence of the condition. For conditions where prolonged remission or cure is possible (i.e. cancer, chronic back pain, depression, severe mental health), additional criteria in relation to duration of disease and/or current treatment were specified, similar to the approach taken by Barnett et al. [30]. Primary care practices were provided with study materials (including participant information sheet, survey pack, pre-paid reply envelope). They selected eligible patients from their practice database according to the inclusion criteria, and mailed the questionnaire packs to 2983 potential participants.

### Questionnaires

The survey included a self-efficacy scale [31, 32], the Long-Term Conditions Questionnaire (LTCQ) [27], the EuroQol 5 Dimension 5 Level (EQ-5D-5L) [33, 34], the Disease Burden Impact Scale (DBIS) [12, 35], and demographics questions. The deprivation score for each participating general practitioner (GP) practice was derived from https://tools.npeu.ox.ac.uk/imd/ (1st May 2018) and converted into quintiles. This information was entered into SPSS for each participant.

Self-efficacy was assessed by the 6-item Self-efficacy for managing Chronic Disease Scale [31, 32]. Each item is rated on a 1 ‘not confident at all’ to 10 ‘totally confident’ scale. The score is the mean of the items, with the score range 1–10. A higher score indicates higher self-efficacy or more confidence in managing chronic disease(s).

Quality of life was assessed by the LTCQ and the EQ-5D-5L. The LTCQ is a 20-item measure that addresses the concept of ‘living well with long-term conditions’. It has been found valid and reliable in health and social care users [27]. The LTCQ was specifically developed to assess outcomes in people with a range of long-term conditions (including physical and mental health conditions), and single and multiple morbidities. Items are scored on a 5 -point scale from ‘Never’ to ‘Always’. A single score is calculated from the 20 items, with scores ranging from 0 to 100 and higher scores indicating ‘living well’.

The EQ-5D-5L [33, 34] is a generic measure of health status that includes five questions covering mobility, self-care, usual activities, pain and depression/anxiety, and a Visual Analogue Scale (EQ-VAS). Each question has five response options where 1 is ‘having no problem’s and 5 is ‘being unable to do the activity’ or ‘extreme pain or anxiety/depression’. The EQ-5D-5L score, calculated from the five questions, has a theoretical range of − 0.285 (a state worse than death) to 1 (best possible health state) [36]. The EQ-VAS measures overall health on the day of completion of the questionnaire and the score ranges from 0 (the worst health you can imagine) to 100 (the best health you can imagine).

The DBIS [12, 35] assesses the personal disease burden of chronic long-term conditions. Developed specifically for primary care, it asks participants to self-report their chronic condition(s), and in a second step to give a rating of the degree to which each condition interferes with daily activities. The original questionnaire includes 21 conditions that are rated on a six point scale where ‘0’ means that a participant does not have the condition, and 1 (none) to 5 (high) to indicate the degree of interference of a condition. The 21 conditions in the original DBIS were all physical health conditions and, as it is permitted by the original developers to add further conditions [12], four further groups were added: MS, depression or anxiety, bipolar disorder, and psychosis or schizophrenia. Therefore, the DBIS in this study included 25 conditions. Space was also provided for additional conditions not already listed, and participants added up to three further conditions. This means that the disease burden score range for this study was 0 (indicating not having any chronic conditions) to 140, with a higher score representing a higher disease burden.

### Analysis

All data were entered into SPSS (version 22), a statistical software package. The self-efficacy, LTCQ, EQ-5D-5L and disease burden (DBIS) scores were calculated according to the developers’ instructions. For self-efficacy, the score can be calculated if at least four out of the six items have been completed (i.e. 837 (98.7%) / 848 participants). For the LTCQ score, 76 (8.9%) cases had missing data and for the EQ-5D-5L, the number of cases with missing data was 20 (2.4%) for the EQ-5D-5L score and 6 (0.7%) for EQ-VAS. No data imputation was undertaken for the LTCQ score, EQ-5D-5L score or the EQ-VAS.

For the disease burden or the DBIS score, it was assumed that if there was no response for a given condition that the participant did not have this condition (ie coded as 0) according to the method by Ramon-Roquin et al. [37]. The conditions added under ‘other’ (open text box) also required some recoding, for example if the ‘other’ condition was one of the 25 conditions listed. If conditions were listed twice through the use of the ‘other’ box, the worst impact score was retained, or if the same impact score was reported, only one score was retained. Based on the open text answers, two additional categories were created ‘other mental health’ and ‘other neurological’ to cover mental health or neurological conditions not in the list of 25 conditions, such as eating disorders, obsessive compulsive disorder or neurological conditions other than MS. After the calculation of the score, 19 participants had a score of 0, indicating that they have none of the conditions listed and 50 reported only one condition. These 69 participants were removed from the analysis as a minimum of two conditions need to co-exist to meet the definition of multi-morbidity.

Descriptive statistics were used to report the sample characteristics, and self-efficacy, LTCQ, EQ-5D-5L, EQ-VAS and DBIS scores. Analysis of Variance (ANOVA) was used to examine the relationship between demographics, presence of a mental health problem, GP practice deprivation score and hospital admission for chronic disease in the last 12 months to the DBIS, LTCQ, EQ-5D-5L, EQ-VAS and self-efficacy scores respectively. ANOVA was also used to examine the relationships between DBIS and LTCQ, EQ-5D-5L scores and EQ-VAS, and the relationship between the presence/absence of individual conditions on the self-efficacy score. Multiple linear regression analyses were conducted to examine the relationship between quality of life (dependent variables LTCQ, EQ-5D-5L and EQ-VAS scores) and self-efficacy, burden of disease (DBIS score), demographics, presence of mental health problem, deprivation score and hospital admission. The level of significance was set at *p* < 0.05. Exact values for p are reported for values ≥0.001, otherwise they are reported as *p* < 0.001.

## Results

### Participants

The total sample size was 848 primary care patients with multi-morbidity, with a slightly larger proportion of respondents being female. The mean age of participants was 67.0 (SD 13.93). The majority were married (*n* = 505, 59.6%) and of a white ethnic background (*n* = 813, 95.9%). One hundred and fourteen (13.6%) of respondents had been admitted to hospital for a chronic condition in the year preceding the study. Further demographic details and health related information can be found in Table 1.

**Table 1:** Demographics and health information

Variable	Response options	*n*	%
Gender	Male	395	46.6
	Female	432	50.9
Age (years)	18–29	14	1.7
	30–39	25	3.1
	40–49	61	7.5
	50–59	99	12.2
	60–69	229	28.3
	70–79	229	28.3
	80–89	141	17.4
	90+	11	1.4
Marital status	Married/living as married/civil partnership	505	59.6
	Separated/divorced	111	13.1
	Widowed	139	16.4
	Single	70	8.3
Ethnicity	White	813	95.9
	Other	11	1.4
Employment ^a^	Employed (full- or part-time)	170	20.0
	Retired	418	49.3
	Permanently sick or disabled	93	11.0
	Other ^b^	93	11.0
IMD quintiles ^c^ of GP practice	1 - Least deprived	191	22.7
	2	285	33.9
	3	51	6.1
	4	167	19.9
	5 - Most deprived	147	17.5
Physical long-term chronic condition	No	6	0.7
	Yes	842	99.3
Mental health condition	No	514	60.6
	Yes	334	39.4
Hospital admission for long-term chronic condition in last year	No	725	86.4
	Yes	114	13.6

NB. The percentages do not always add up to 100% due to missing data

^a^Data were coded missing for 74 (8.7%) respondents, either as the question had not been answered (*n* = 23, 2.7%) or as multiple answers had been given when only one response was permitted (*n* = 51, 6.0%)

^b^Includes full or part-time education, unemployed, looking after the home, voluntary or charity work and doing something else. These categories were collapsed as each applied to less than 5% of the sample

^c^IMD – Index of multiple deprivation quintiles of participants’ GP practice

### Chronic conditions and disease burden

The mean number of LTCs reported was 6.46 (SD 3.49), the mean number of physical LTCs was 5.99 (SD 3.34) and mental health conditions 0.47 (SD 0.66). All but 6 respondents reported at least one physical health condition, and 334 (39.4%) reported at least one mental health condition. The most commonly reported conditions were hypertension, problems with vision and being overweight (Table 2). The mean disease burden (DBIS) score was 15.45 (SD 12.00). The disease burden score was significantly different by employment (*p* < 0.001), marital status (*p* = 0.029), presence of a mental health problem (*p* < 0.001), deprivation score of the GP practice (*p* < 0.001), and hospital admission in the last year (*p* < 0.001).

**Table 2:** Prevalence of each long-term chronic condition and mean self-efficacy (and standard deviation (SD)) by presence or absence of each condition

Condition	Participants with the condition			Self-efficacy			
		*n*	%	*n*	Mean	SD	*p*
Hypertension ^a^	*Yes*	435	51.3	431	6.78	2.60	0.27
	*No*	413	48.7	406	6.59	2.47	
Problems with vision ^a^	*Yes*	396	46.7	392	7.07	2.50	< 0.001
	*No*	452	53.3	445	6.26	2.52	
Overweight ^a^	*Yes*	379	44.7	377	6.14	2.36	< 0.001
	*No*	469	55.3	460	7.14	2.36	
Back pain or sciatica ^a^	*Yes*	334	39.4	329	5.53	2.45	< 0.001
	*No*	514	60.6	508	7.44	2.30	
Depression ^a^	*Yes*	326	38.4	325	5.24	2.43	< 0.001
	*No*	522	61.6	512	7.61	2.15	
Problems with hearing ^a^	*Yes*	320	37.7	317	6.41	2.58	0.014
	*No*	528	62.3	520	6.86	2.52	
Circulation problems in legs ^a^	*Yes*	307	36.2	301	5.69	2.47	< 0.001
	*No*	541	63.8	536	7.25	2.41	
Cholesterol ^a^	*Yes*	285	45.4	381	6.60	2.65	0.35
	*No*	463	54.6	456	6.76	2.44	
Diabetes ^a^	*Yes*	251	29.6	248	6.98	2.75	0.035
	*No*	597	70.4	589	6.57	2.49	
Stomach problems ^a^	*Yes*	244	28.8	241	5.79	2.45	< 0.001
	*No*	604	71.2	596	7.05	2.55	
Osteoarthritis ^a^	*Yes*	234	27.6	231	5.96	2.52	< 0.001
	*No*	614	72.4	606	6.97	2.49	
Colon problems ^a^	*Yes*	225	26.5	224	5.78	2.58	< 0.001
	*No*	623	73.5	613	7.02	2.44	
Heart disease ^a^	*Yes*	222	26.2	217	6.57	2.54	0.41
	*No*	626	73.8	620	6.73	2.54	
Asthma ^a^	*Yes*	164	80.6	161	5.80	2.65	< 0.001
	*No*	684	19.3	676	6.90	2.68	
COPD ^a^	*Yes*	146	17.2	144	5.99	2.57	< 0.001
	*No*	702	82.8	693	6.84	2.51	
Rheumatoid arthritis ^a^	*Yes*	134	15.8	133	5.66	2.48	< 0.001
	*No*	714	84.2	704	6.88	2.50	
Cancer ^a^	*Yes*	130	15.3	129	6.67	2.57	0.91
	*No*	718	87.6	708	6.69	2.54	
Stroke ^a^	*Yes*	122	14.4	115	6.32	2.50	0.093
	*No*	726	85.6	722	6.75	2.54	
Thyroid problems ^a^	*Yes*	119	14.0	116	6.06	2.62	0.004
	*No*	729	86.0	721	6.79	2.51	
Osteoporosis ^a^	*Yes*	105	12.4	231	5.96	2.52	< 0.001
	*No*	743	87.6	606	6.99	2.49	
Rheumatic disease ^a^	*Yes*	78	9.2	78	4.89	2.67	< 0.001
	*No*	770	15.8	759	6.88	2.45	
Congestive heart failure ^a^	*Yes*	67	7.9	67	5.93	2.43	0.011
	*No*	781	92.1	770	6.76	2.54	
Multiple sclerosis ^b^	*Yes*	37	4.4	37	5.30	2.53	0.001
	*No*	811	95.6	800	6.75	2.52	
Bipolar disorder ^b^	*Yes*	30	3.5	30	5.10	2.44	< 0.001
	*No*	818	96.5	807	6.75	2.53	
Psychosis or Schizophrenia ^b^	*Yes*	29	3.4	29	4.99	2.49	< 0.001
	*No*	819	96.6	808	6.75	2.52	
Other neurological ^c^	*Yes*	24	2.8	24	5.41	2.04	0.012
	*No*	824	97.2	813	6.73	2.54	
Other mental health ^c^	*Yes*	10	1.2	10	4.42	2.61	0.004
	*No*	838	98.8	827	6.71	2.53	
Other 1	*Yes*	171	20.2				
	*No*	677	79.8				
Other 2	*Yes*	42	5.0				
	*No*	806	95.0				
Other 3	*Yes*	8	0.9				
	*No*	840	99.1				

^a^One of the 21 conditions listed in the original DBIS (plus space to add additional ‘other’ conditions)

^b^Added for this study

^c^Computed for this study from responses written in ‘other’

NB. Self-efficacy was measured by the Self-efficacy for managing Chronic Disease Scale (score range 1–10, with higher scores indicating better self-efficacy)

### Quality of life and well-being scores

The mean scores for the EQ-5D -5L was 0.69 (SD 0.28), the EQ-VAS 65.44 (SD 23.66) and the LTCQ 69.31 (SD 21.77). For the EQ-5D-5L score, significant differences were found for gender (*p* = 0.022), employment (*p* < 0.001), marital status (*p* < 0.001), the presence of a mental health problem (*p* < 0.001), the DBIS (*p* < 0.001) and deprivation score of the GP practice (*p* < 0.001). The EQ-VAS was significantly different for gender (*p* = 0.043), age (*p* = 0.026), employment status (*p* < 0.001), marital status (p < 0.001), presence of a mental health condition (*p* < 0.001), the DBIS (*p* < 0.001), and deprivation score of the respondent’s GP practice (*p* < 0.001). The LTCQ score was significantly different for gender (*p* = 0.001), age (*p* < 0.001), marital status (*p* < 0.001), employment (*p* < 0.001), presence of mental health condition (*p* < 0.001), the DBIS (*p* < 0.001) and the deprivation score of the GP practice (*p* < 0.001). (Tables with mean scores, standard deviation and level of significance can be found in Additional file 1).

### Self-efficacy

**Fig. 1:**
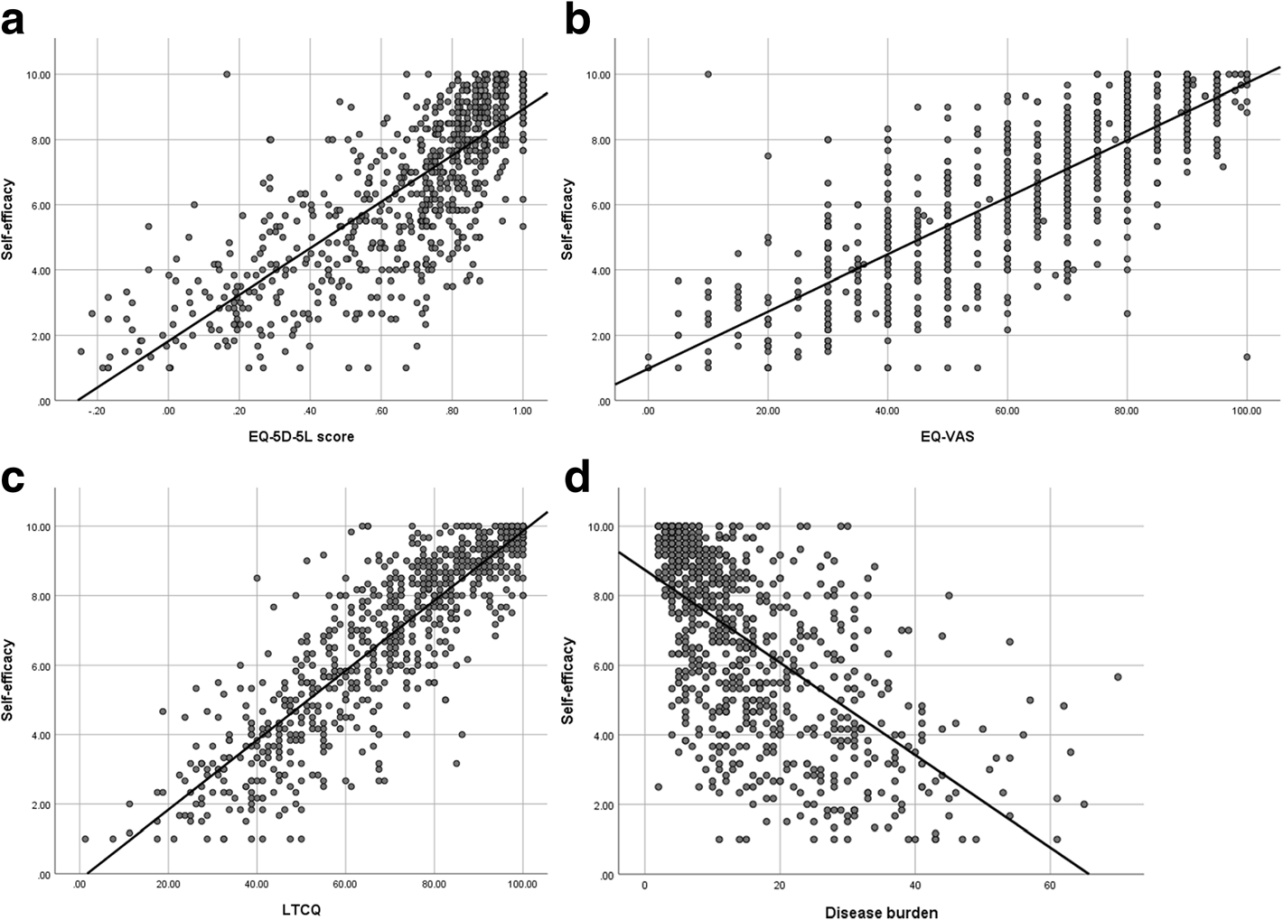
Self-efficacy (measured by the Self-efficacy for managing Chronic Disease Scale) by EQ-5D-5L score (**a**), EQ-VAS (**b**), Long-term Condition Questionnaire (LTCQ) (**c**) and disease burden (measured by the Disease Burden Impact Scale) (**d**)

The mean self-efficacy score for the total sample (*n* = 837) was 6.69 (SD 2.53). Self-efficacy was significantly different for gender (*p* = 0.007), age (*p* = 0.001), employment (*p* < 0.001), marital status (p < 0.001), presence of a mental health condition (*p* < 0.001), and in those registered at a GP practice in a more deprived area (*p* < 0.001). There was no significant difference for ethnicity. Presence of a physical health problem did not show any significant differences in self-efficacy, but there were only 6 people in the sample who did not report a physical health condition. Many of the self-reported long term conditions were associated with lower self-efficacy (see Table 2). Self-efficacy was lower in participants reporting increasing disease burden (*p* < 0.001) and those reporting lower EQ-5D-5 L scores, lower EQ-VAS and lower LTCQ scores (all p < 0.001). The relationships between self-efficacy and EQ-5D-5 L, EQ-VAS, LTCQ and disease burden are illustrated in Fig. 1. (Tables with mean scores, standard deviation and level of significance can be found in Additional file 1).

### Regression analysis

Linear regression was used to examine the impact of self-efficacy, controlled for disease burden, other disease related factors, and demographics on quality of life (EQ-5D-5L (Table 3), EQ-VAS (Table 4) and LTCQ (Table 5) of these primary care patients with multi-morbidity. All three models were statistically significant (all *p* < 0.001, with strong adjusted R^2^ of > 0.70). Significant factors in all models, in addition to self-efficacy, were disease burden (DBIS score) and being permanently sick or disabled. Other factors varied between models, with the most notable being the presence of a mental health condition in the LTCQ model.

**Table 3:** Linear regression for EQ-5D-5L (dependent variable) and self-efficacy (measured by the Self-efficacy for Managing Chronic Disease Scale) controlled for disease burden and demographics (*p* < 0.001, adjusted R^2^ = 0.70)

Variables	Standardized Coefficients	t	95% Confidence interval		*p*
			Lower	Upper	
(Constant)		0.29	0.48	7.62	< 0.001
Self-efficacy	0.54	0.06	0.07	19.33	< 0.001
Disease burden	− 0.24	− 0.01	− 0.004	− 9.05	< 0.001
Gender	− 0.02	− 0.03	0.01	− 0.88	0.38
Age	0.05	0.00	0.002	1.83	0.07
Separated or divorced	−0.02	− 0.05	0.02	− 0.85	0.40
Widowed	−0.01	−0.04	0.03	−0.39	0.70
Single	0.01	− 0.03	0.05	0.42	0.67
Retired	−0.08	−0.08	− 0.01	−2.89	0.004
Permanently sick/disabled	− 0.19	− 0.21	− 0.12	−7.62	< 0.001
Other occupation	0.01	−0.03	0.06	0.50	0.62
GP practice IMD quintile	−0.03	−0.01	0.002	−1.58	0.12
Mental health condition (yes/no)	−0.02	− 0.04	0.02	− 0.63	0.53
Hospital admission for long-term chronic conditon	−0.05	−0.08	− 0.01	−2.67	0.008

*IMD* Index of multiple deprivation

**Table 4:** Linear regression for EQ-VAS (dependent variable) and self-efficacy (measured by the Self-efficacy for Managing Chronic Disease Scale) controlled for disease burden and demographics (*p* < 0.001, adjusted R^2^ = 0.71)

Variables	Standardized Coefficient	t	95% Confidence interval		*p*
			Lower	Upper	
(Constant)		16.41	33.42	5.75	< 0.001
Self-efficacy	0.68	5.83	6.88	23.73	< 0.001
Disease burden	−0.14	−0.04	−0.18	−5.27	< 0.001
Gender	−0.01	−2.31	1.69	−0.30	0.76
Age	0.02	−0.06	0.13	0.72	0.47
Separated/divorced	−0.01	−3.40	2.39	−0.34	0.73
Widowed	0.03	−0.98	4.68	1.28	0.20
Single	−0.03	−6.51	0.74	−1.56	0.12
Retired	0.01	−1.98	3.26	0.48	0.63
Permanently sick/disabled	−0.08	−9.31	−2.04	−3.06	0.002
Other occupation	0.05	0.06	7.55	2.00	0.046
GP practice IMD quintile	0.004	−0.60	0.72	0.18	0.86
Presence of mental health condiction	0.03	−0.75	3.79	1.31	0.19
Hospital admission for long-term chronic condition	−0.05	−5.87	−0.25	−2.14	0.033

*IMD* Index of multiple deprivation

**Table 5:** Linear regression for LTCQ (dependent variable) and self-efficacy (measured by the Self-efficacy for Managing Chronic Disease Scale) controlled for disease burden and demographics (*p* < 0.001, adjusted R^2^ = 0.78)

Variables	Standardized Coefficients	t	95% Confidence interval		*p*
			Lower	Upper	
(Constant)		19.07	32.64	7.48	< 0.001
Self-efficacy	0.71	5.78	6.63	28.55	< 0.001
Disease burden	−0.11	−0.29	−0.12	−4.63	< 0.001
Gender	0.00	−1.62	1.58	−0.02	0.98
Age	0.08	0.05	0.20	3.18	0.002
Separated/divorced	−0.01	−2.63	1.99	−0.27	0.79
Widowed	−0.03	− 4.05	0.50	−1.53	0.13
Single	−0.04	−6.31	−0.57	−2.35	0.02
Retired	0.01	−1.66	2.51	0.40	0.69
Permanently sick/disabled	−0.07	−8.06	−2.25	−3.49	0.001
Other occupation	−0.004	−3.29	2.72	−0.19	0.85
GP practice IMD quintile	−0.04	−1.10	−0.05	−2.13	0.03
Mental health condition (yes/no)	−0.06	−4.51	−0.88	−2.92	0.004
Hospital admission for long-term chronic condition	−0.01	−2.84	1.54	−0.58	0.56

*IMD* Index of multiple deprivation

## Discussion

Primary care patients with multi-morbidity in England experience lower quality of life if their self-efficacy, i.e. their confidence to manage their diseases, is lower. Furthermore, they experience higher personal burden of disease when they reported lower self-efficacy. This is similar to previous US studies, which also found lower self-efficacy with higher disease burden in multi-morbidity [12, 38]. Differences in self-efficacy were found for the majority of conditions (i.e. whether a specific disease was reported or not), although no significant differences were found for some diseases including cancer, stroke and high cholesterol. To the best of our knowledge, there is no evidence that may explain these differences, and further research is needed to understand differences in self-efficacy between different types of chronic long-term conditions.

The mean self-efficacy in this study was 6.69 (SD 2.53), which was higher than the 5.17 reported in the original study on the self-efficacy scale [31]. Although some studies (e.g. [12, 38]) report the impact of self-efficacy on disease burden or quality of life in multi-morbidity, they do not report mean levels of self-efficacy nor factors associated with self-efficacy such as the demographic or disease-related factors reported here. A German study on multi-morbidity, using the same self-efficacy scale used in this study, reported a similar mean self-efficacy of 6.69 (SD 2.32) [39].

The regression models used to investigate the impact of self-efficacy controlled for disease burden; other disease-related factors and demographics on quality of life were highly significant and also showed that lower self-efficacy was related to lower quality of life. There is evidence that a more person-centred care approach can enhance self-efficacy in single diseases (e.g. acute coronary syndrome [40], stroke [41]) and that self-management support in chronic diseases improves self-efficacy and patient outcomes [31]. As self-efficacy is modifiable, patients with multi-morbidity could experience better quality of life, and services may benefit from a reduction in use through effective self-management support by health care professionals. Enhanced self-efficacy and self-management may be achieved through teaching transferrable disease management skills to patient with multi-morbidity. A lay-led intervention, which involved teaching sessions on relaxation, diet, exercise, fatigue, breaking the “symptom cycle”, managing pain and medication, and communication, led to significantly enhanced self-efficacy [42]. More patient-centred communication during consultations has been shown to be associated with higher self-efficacy [38]. Health professionals should be encouraged to be aware of levels of self-efficacy of their patients with multi-morbidity as it will enable them to support patients more effectively. For example it has been shown in COPD patients that better understanding of illness leads to higher self-efficacy [43]. Other approaches to greater awareness of self-efficacy could range from health professionals asking questions on self-efficacy during consultations to more formal use of outcome measures such as those used in this study.

Some limitations of this study need to be acknowledged. The response rate was 31%, with 3% of those respondents needing to be excluded from this analysis. Response rates in primary care surveys in England have ranged from 15.9 to 38% [7, 44, 45], and hence the response rate in this study was not unusual. However it does mean that the results need to be interpreted with caution as they may not be representative for all primary care patients with multi-morbidity. The study is based on a cross-sectional design and thus it is not possible to establish cause and effect between quality of life and self-efficacy. Although it is generally accepted that self-efficacy is a modifiable factor that can enhance quality of life, clinical trials are necessary to provide definitive answers on causality. Finally, the combination of diseases may also have an impact on self-efficacy, but this was not investigated beyond the impact of the co-existence of a mental health problem with a physical problem (the presence of a mental health condition significantly lowers self-efficacy) and should be investigated further. Studies that have investigated clustering of diseases have found different clusters, for example Deruaz-Luyet et al. [46] identified four clusters including (1) cardiovascular risk factors and conditions, (2) general age-related and metabolic conditions, (3) tobacco and alcohol dependencies, and (4) pain, musculoskeletal and psychological conditions. On the other hand, Schafer et al. [47] identified three clusters 1) cardiovascular/metabolic, 2) anxiety/depression/somatoform disorder and pain, and 3) neuropsychiatric disorders. More evidence is needed on how to best cluster diseases for this type of analysis, but it is an interesting area for future research.

## Conclusion

Multi-morbidity is increasing, and self-management is considered essential for its effective management. This study shows that primary care patients with lower self-efficacy and higher disease burden have lower quality of life. Awareness of health professionals of self-efficacy of their patients with multi-morbidity would help to identify patients who are in need of enhanced self-management support. Providing self-management support for chronic disease has been hailed as a hallmark of good care [48]. Higher self-efficacy may lead to enhanced quality of life for people with multi-morbidity.

## Additional file

Additional file 1: **Table S1.** Mean EQ-5D-5L scores (with a theoretical range of − 0.285 (a state worse than death) to 1 (best possible health state) by demographic and disease-related variables. **Table S2.** Mean EQ-VAS scores (range 1–100, with higher scores indicating better health) by demographic and disease-related variables. **Table S3.** Mean LTCQ scores (range 0–100, with higher scores indicating ‘living well’) by demographic and disease-related variables. **Table S4.** Self-efficacy score (measured by the Self-efficacy for Managing Chronic Disease Scale with a score range of 1–10, with higher scores indicating higher self-efficacy) by participants’ characteristics. (DOCX 29 kb)

## Abbreviations

ANOVA: Analysis of variance
COPD: Chronic obstructive pulmonary disease
DBIS: Disease burden impact scale
EQ-5D-5L: EuroQOL 5 dimension 5 level
EQ-VAS: EuroQOL visual analogue scale
GP: General practitioner
IBS: Irritable bowel syndrome
IHD: Ischaemic heart disease
IMD: Index of multiple deprivation
LTCQ: Long-Term Conditions Questionnaire
MS: Multiple sclerosis
NHS: National Health Service
OA: Osteoarthritis
