# Supplementary material for: Self-efficacy and health-related quality of life: a cross-sectional study of primary care patients with multi-morbidity
Source: Health Qual Life Outcomes. 2019 Feb 14;17:37. doi: 10.1186/s12955-019-1103-3 (PMC6376655; doi:10.1186/s12955-019-1103-3)
Supplement: Supplementary file 1 — Table S1. Mean EQ-5D-5L scores (with a theoretical range of − 0.285 (a state worse than death) to 1 (best possible health state) by demographic and disease-related variables. Table S2. Mean EQ-VAS scores (range 1–100, with higher scores indicating better health) by demographic and disease-related variables. Table S3. Mean LTCQ scores (range 0–100, with higher scores indicating ‘living well’) by demographic and disease-related variables. Table S4. Self-efficacy score (measured by the Self-efficacy for Managing Chronic Disease Scale with a score range of 1–10, with higher scores indicating higher self-efficacy) by participants’ characteristics. (DOCX 29 kb) [file 12955_2019_1103_MOESM1_ESM.docx]

# Additional file 1

Tables S1-S3: EQ-5D-5L, EQ-VAS and LTCQ scores by demographics and disease-related variables

Table S4: Self-efficacy by demographics and disease-related variables

**Table S1: Mean EQ-5D-5L scores (with a theoretical range of -0.285 (a state worse than death) to 1 (best possible health state) by demographic and disease-related variables**

| **Variable** | **Response options** | **N** | **Mean** | **SD** | **p** |
| --- | --- | --- | --- | --- | --- |
| **Gender** | *Male* | 389 | 0.72 | 0.27 | 0.022 |
|  | *Female* | 419 | 0.67 | 0.29 |  |
| **Age ^1^** | *18-29 years* | 13 | 0.70 | 0.22 | 0.001 |
|  | *30-39 years* | 25 | 0.63 | 0.28 |  |
|  | *40-49 years* | 60 | 0.61 | 0.32 |  |
|  | *50-59 years* | 96 | 0.63 | 0.35 |  |
|  | *60-69 years* | 229 | 0.67 | 0.30 |  |
|  | *70-79 years* | 223 | 0.75 | 0.25 |  |
|  | *80-89 years* | 137 | 0.72 | 0.22 |  |
|  | *90+ years* | 8 | 0.74 | 0.15 |  |
| **Employment** | *Employed (Full-time or part-time* | 168 | 0.77 | 0.19 | <0.001 |
|  | *Retired* | 410 | 0.73 | 0.25 |  |
|  | *Permanently sick or disabled* | 91 | 0.31 | 0.29 |  |
|  | *Other* | 91 | 0.77 | 0.22 |  |
| **Marital status** | *Married/ Living as married/ Civil partnership* | 494 | 0.71 | 0.27 | <0.001 |
|  | *Separated/ Divorced* | 108 | 0.58 | 0.35 |  |
|  | *Widowed* | 136 | 0.73 | 0.25 |  |
|  | *Single* | 68 | 0.66 | 0.30 |  |
| **IMD quintile ^2^** | *Least deprived* | 184 | 0.77 | 0.23 | <0.001 |
|  | *2* | 281 | 0.73 | 0.25 |  |
|  | *3* | 51 | 0.67 | 0.29 |  |
|  | *4* | 162 | 0.59 | 0.33 |  |
|  | *Most deprived* | 144 | 0.64 | 0.31 |  |
| **Mental health problem** | *No* | 503 | 0.78 | 0.23 | <0.001 |
|  | *Yes* | 325 | 0.56 | 0.31 |  |
| **Hospital admission last 12 months for long-term chronic condition** | *No* | 707 | 0.72 | 0.27 | <0.001 |
|  | *Yes* | 112 | 0.53 | 0.28 |  |

^1^ Age groups created for ease of presentation within this table. Age is used as a continuous variable within the main body of the paper.

^2^ IMD – Index of multiple deprivation quintiles of participants’ GP practice

**Table S2: Mean EQ-VAS scores (range 1-100, with higher scores indicating better health) by demographic and disease-related variables**

| **Variable** | **Response options** | **N** | **Mean** | **SD** | **p** |
| --- | --- | --- | --- | --- | --- |
| **Gender** | *Male* | 394 | 67.08 | 22.90 | 0.043 |
|  | *Female* | 428 | 63.76 | 24.08 |  |
| **Age ^1^** | *18-29 years* | 14 | 59.57 | 15.59 | <0.001 |
|  | *30-39 years* | 25 | 58.20 | 25.99 |  |
|  | *40-49 years* | 61 | 55.93 | 24.57 |  |
|  | *50-59 years* | 99 | 59.11 | 26.90 |  |
|  | *60-69 years* | 227 | 65.39 | 24.44 |  |
|  | *70-79 years* | 228 | 70.50 | 21.34 |  |
|  | *80-89 years* | 139 | 67.78 | 20.67 |  |
|  | *90+ years* | 11 | 70.46 | 14.05 |  |
| **Employment** | *Employed (full-time or part-time)* | 169 | 69.08 | 20.75 | 0.001 |
|  | *Retired* | 414 | 69.95 | 21.25 |  |
|  | *Permanently sick or disabled* | 93 | 36.24 | 18.04 |  |
|  | *Other* | 93 | 69.70 | 20.97 |  |
| **Marital status** | *Married/ Living as married/ Civil partnership* | 502 | 66.27 | 23.02 | <0.001 |
|  | *Separated/ Divorced* | 111 | 56.62 | 25.44 |  |
|  | *Widowed* | 137 | 71.69 | 20.64 |  |
|  | *Single* | 70 | 59.97 | 25.44 |  |
| **IMD quintile ^2^** | *Least deprived* | 189 | 69.84 | 21.77 | <0.001 |
|  | *2* | 283 | 68.51 | 21.99 |  |
|  | *3* | 51 | 64.35 | 24.98 |  |
|  | *4* | 166 | 59.57 | 25.87 |  |
|  | *Most deprived* | 146 | 61.28 | 24.04 |  |
| **Mental health problem** | *No* | 509 | 72.50 | 20.81 | <0.001 |
|  | *Yes* | 333 | 54.64 | 23.69 |  |
| **Hospital admission last 12 months for long-term chronic condition** | *No* | 721 | 67.49 | 22.8 | <0.001 |
|  | *Yes* | 112 | 53.49 | 25.21 |  |

^1^ Age groups created for ease of presentation within this table. Age is used as a continuous variable within the main body of the paper.

^2^ IMD – Index of multiple deprivation quintiles of participants’ GP practice

**Table S3: Mean LTCQ scores (range 0-100, with higher scores indicating ‘living well’) by demographic and disease-related variables**

| **Variable** | **Response options** | **N** | **Mean** | **SD** | **p** |
| --- | --- | --- | --- | --- | --- |
| **Gender** | *Male* | 361 | 72.07 | 20.89 | 0.001 |
|  | *Female* | 394 | 66.58 | 22.33 |  |
| **Age ^1^** | *18-29 years* | 12 | 52.19 | 19.89 | <0.001 |
|  | *30-39 years* | 25 | 58.25 | 22.60 |  |
|  | *40-49 years* | 59 | 58.26 | 22.38 |  |
|  | *50-59 years* | 95 | 61.01 | 24.42 |  |
|  | *60-69 years* | 214 | 70.08 | 21.01 |  |
|  | *70-79 years* | 207 | 75.96 | 19.61 |  |
|  | *80-89 years* | 118 | 72.27 | 18.98 |  |
|  | *90+ years* | 9 | 68.89 | 13.54 |  |
| **Employment** | *Employed (Full-time or part-time)* | 162 | 72.28 | 18.26 | 0.001 |
|  | *Retired* | 378 | 74.99 | 19.37 |  |
|  | *Permanently sick or disabled* | 85 | 40.44 | 14.63 |  |
|  | *Other* | 85 | 69.31 | 20.20 |  |
| **Marital status** | *Married/ Living as married/ Civil partnership* | 466 | 70.80 | 21.18 | 0.001 |
|  | *Separated/ Divorced* | 101 | 61.00 | 23.94 |  |
|  | *Widowed* | 123 | 73.73 | 19.13 |  |
|  | *Single* | 63 | 61.79 | 23.10 |  |
| **IMD quintile ^2^** | *Least deprived* | 177 | 75.54 | 19.66 | <0.001 |
|  | *2* | 262 | 72.24 | 19.37 |  |
|  | *3* | 47 | 67.50 | 20.18 |  |
|  | *4* | 152 | 62.13 | 24.48 |  |
|  | *Most deprived* | 130 | 64.47 | 22.79 |  |
| **Mental health problem** | *No* | 470 | 77.52 | 18.01 | <0.001 |
|  | *Yes* | 302 | 56.54 | 20.95 |  |
| **Hospital admission last 12 months for long-term chronic condition** | *No* | 660 | 71.10 | 21.52 | <0.001 |
|  | *Yes* | 105 | 59.70 | 20.60 |  |

^1^ Age groups created for ease of presentation within this table. Age is used as a continuous variable within the main body of the paper.

^2^ IMD – Index of multiple deprivation quintiles of participants’ GP practice

**Table S4: Self-efficacy score (measured by the Self-efficacy for managing Chronic Disease Scale with a score range of 1-10, with higher scores indicating higher self-efficacy) by participants’ characteristics**

| **Variable** | **Response options** | **N** | **Mean** | **SD** | **p** |
| --- | --- | --- | --- | --- | --- |
| **Gender** | *Male* | 389 | 6.94 | 2.45 | 0.007 |
|  | *Female* | 428 | 6.46 | 2.59 |  |
| **Age ^1^** | *18-29 years* | 14 | 5.64 | 1.98 | <0.001 |
|  | *30-39 years* | 25 | 5.77 | 2.34 |  |
|  | *40-49 years* | 61 | 5.66 | 2.36 |  |
|  | *50-59 years* | 99 | 5.90 | 2.72 |  |
|  | *60-69 years* | 228 | 6.79 | 2.53 |  |
|  | *70-79 years* | 224 | 7.26 | 2.50 |  |
|  | *80-89 years* | 138 | 6.88 | 2.36 |  |
|  | *90+ years* | 11 | 6.82 | 2.12 |  |
| **Employment** | *Employed (Full-time or part-time)* | 169 | 7.14 | 2.07 | <0.001 |
|  | *Retired* | 413 | 7.19 | 2.39 |  |
|  | *Permanently sick or disabled* | 92 | 3.65 | 1.80 |  |
|  | *Other* | 92 | 6.91 | 2.40 |  |
| **Marital status** | *Married/ Living as married/ Civil partnership* | 497 | 6.79 | 2.52 | <0.001 |
|  | *Separated/ Divorced* | 111 | 5.72 | 2.65 |  |
|  | *Widowed* | 138 | 7.26 | 2.28 |  |
|  | *Single* | 69 | 6.34 | 2.51 |  |
| **IMD quintile ^2^** | *Least deprived* | 190 | 7.25 | 2.34 | <0.001 |
|  | *2* | 283 | 7.00 | 2.33 |  |
|  | *3* | 50 | 6.59 | 2.56 |  |
|  | *4* | 164 | 6.03 | 2.74 |  |
|  | *Most deprived* | 143 | 6.21 | 2.66 |  |
| **Mental health problem** | *No* | 504 | 7.61 | 2.15 | <0.001 |
|  | *Yes* | 333 | 5.29 | 2.44 |  |
| **Hospital admission last 12 months for long-term chronic condition** | *No* | 715 | 6.91 | 2.48 | <0.001 |
|  | *Yes* | 114 | 5.47 | 2.53 |  |

^1^ Age groups created for ease of presentation within this table. Age is used as a continuous variable within the main body of the paper.

^2^ IMD – Index of multiple deprivation quintiles of participants’ GP practice
